# Pharmacotherapy for Borderline Personality Disorder: an Update of Published, Unpublished and Ongoing Studies

**DOI:** 10.1007/s11920-020-01164-1

**Published:** 2020-06-05

**Authors:** Jutta Stoffers-Winterling, Ole Jakob Storebø, Klaus Lieb

**Affiliations:** 1 Department of Psychiatry and Psychotherapy, University Medical Center of the Johannes Gutenberg University Mainz, Untere Zahlbacher Straße 8, D-55131 Mainz, Germany; 2 Center for Evidence-Based Psychiatry, Psychiatric Research Unit, Slagelse, Region Zealand Psychiatry Denmark; 3 Department of Psychology, Faculty of Health Sciences, University of Southern Denmark, Odense, Denmark

**Keywords:** Borderline personality disorder, Drug treatment, Review, Antidepressants, Antipsychotics, Anticonvulsants

## Abstract

**Purpose of the Review:**

We aim to identify the most recent evidence of randomised controlled trials evaluating continued drug treatments in people with a diagnosis of BPD, review the most recent findings, highlight trends in terms of currently ongoing studies and comment on the overall body of evidence.

**Recent Findings:**

We identified seven new RCTs, plus newly available data for an older RCT. Only three of these RCTs have been published in full text, while we found study data posted at trial registry platforms for the others.

**Summary:**

The new findings do not support fluoxetine as a treatment option for suicide and self-harm prevention. A large effectiveness study did not detect beneficial effects of lamotrigine in routine care. The prevalent use of medications in BPD is still not reflected or supported by the current evidence. More research is needed regarding the most commonly used substances and substance classes, i.e. SSRIs, and quetiapine, but also with respect to people presenting with distinct comorbid conditions.

## Introduction

**Fig. 1:**
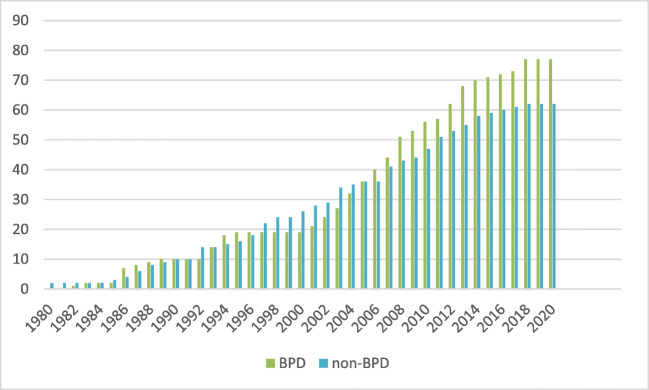
Randomised and quasi-randomised controlled trials of drug treatments in people with personality disorders. Publications per year, 1980 to current; CENTRAL search 29 March 2020 (MeSH descriptor [borderline personality disorder] AND qualifier “drug therapy”)

Considering the numbers of randomised controlled trials (RCTs) testing drug treatments for borderline personality disorder (BPD), it becomes clear that throughout the last 5 years, this topic seems to be paid less attention to (Fig. 1). Even though some late 2019 papers may not yet have been identified in our March 2020 search, the trend is evident: drug treatments are paid less attention to in current research. This observation is in line with recommendations of the major clinical guidelines for BPD, which concordantly recommend psychotherapy as the first-line treatment [1, 2]. It seems that the therapeutic pessimism, which once prevailed for psychotherapeutic interventions in BPD, now holds for drug treatment. This is not surprising giving the rapidly accumulating evidence of psychotherapy evaluation studies in BPD, and encouraging findings: whereas the previous corresponding Cochrane review included 28 randomised controlled trials in 2012 [3], the 2020 update identified 75 such studies [4]. New systematic reviews and meta-analyses support beneficial effects of psychotherapeutic interventions on relevant clinical outcomes such as psychosocial functioning, BPD severity or self-harming and suicidal behaviour [5, 6].

Nevertheless, most people with a diagnosis of BPD receive medications of a large variety of substance classes and oftentimes many drugs concurrently [7, 8]. Reasons are the absence of psychotherapy [9], comorbid conditions requiring medication, or the addition of new substances in times of crisis, which, in the absence of regular medication reviews, simply accumulate over time.

Facing this clinical situation, it seems worthwhile to keep track of research in the field. New substances and even substance classes have been suggested, especially if specific comorbidities like depressive disorders are present. Building on our 2015 review [10], we aim to follow up recent developments in the field of pharmacotherapy for BPD, and critically discuss and integrate the new studies in the existing body of evidence. We will also highlight ongoing research.

## Methods

We focused on randomised controlled trials (RCTs) of drug treatments for people with borderline personality disorder that reported clinical outcomes. We did not consider studies that concentrated on neurobiological outcomes, or which tested the immediate effects of a once-administered substance. We searched the CENTRAL database of the Cochrane Library in March 2020 for relevant publications from 2015 to present. We also traced back references of study trial register records that we had cited as ongoing studies in our 2015 review [10] in order to identify corresponding full publications. Last, we searched trial registers, i.e. the EudraCT register using the term “borderline personality disorder”, the ISRCTN registry (studies with inclusion criterion “borderline personality disorder”), ClinicalTrials.com “Borderline Personality Disorder AND interventional studies”), and the WHO meta-register ICTRP (“borderline personality disorder”).

If study results were posted on trial registries without statistical analysis, we statistically compared treatment groups by calculating either standardised mean differences (SMDs) for continuous outcomes or Risk Ratios (RRs) for dichotomous outcomes from post-treatment values, data permitting.

## Results of the Search

Focusing on publication dates 2015 to current (March 2020), we identified five full-text publications [11•, 12••, 13•, 14•, 15••] reporting on four different RCTs with clinical outcomes. One of these records [14•] reported additional data of the Black et al. 2014 RCT [16•], which had already been included in the 2015 review [10]. Two of these records referred to the same study [12••, 15••]. Searching trial platforms, we identified new study data for five RCTs. One referred also to the Black 2014 RCT [17•], whereas four related to RCTs that have not been published elsewhere [18•, 19•, 20•, 21•]. Table 1 gives an overview of the main characteristics of these studies.

**Table 1:** Randomised-controlled trials of drug treatments for borderline personality disorder published between 2015 and March 2020

RCTs	Participants	Interventional drug	Comparison treatment	Observation period	Outcomes	Effects	
Antidepressants							
NCT00834834 [18]	*n* = 84 participants with BPD (DSM-IV), 8.3% men, suicide attempt within past 2 months, additional suicide attempt(s) or self-injury episode in past 12 months	Fluoxetine (up to 40 mg/day) + clinical management, switching to citalopram (up to 60 mg/day) if necessary	DBT	6 months	Suicidal and suicide-related behaviour, serious and non-serious AE	Significantly more suicide attempts in fluoxetine group (RR 2.87, 95% CI 1.15, 7.20)	
NCT00533117 [19]	*n* = 75 participants with BPD, 22.7% men, at least one suicide attempt or self-mutilation episode 12 months prior to study entry, continued urges to self-mutilate or attempt suicide	Fluoxetine (up to 80 mg/day) + DBT or + SP	Placebo + DBT or + SP	12 months	Suicidal ideation, suicide attempts, and self-mutilation	Data available only for serious AE (including suicide attempts), difference between groups with active drug and placebo n.s.	
Antipsychotics							
Bozzatello et al. 2017 [11]	*n* = 51 outpatients with BPD	Olanzapine (5–10 mg/day)	Asenapine (5–10 mg/day)	12 weeks	Clinical severity, depression, anxiety, psychosocial functioning, BPD symptoms, BPD severity, impulsiveness, aggression, self-harm, and AE	Olanzapine superior: dissociation/paranoid ideation (*η*^*2*^ = 0.21, *p* = 0.012), asenapine superior: affective instability (*η*^*2*^ = 0.53, *p* = 0.001)	
Black 2014 [16]: new final data posted at ClinicalTrials.gov [17], additional data published in paper by Lee et al. [14]	*n* = 97 outpatients with BPD, 29.5% men (common comorbidities were excluded: MDD, PTSD, panic disorder, obsessive-compulsive disorder, substance dependence or abuse (other than alcohol/nicotine)	Quetiapine 150 mg/day(*n* = 33); quetiapine 300 mg/day (*n* = 33)	Placebo (*n* = 29)	8 weeks	ClinicalTrials.gov [17]: BPD severity, psychosocial functioning, impulsiveness, aggression, mania, depression, general psychopathology, and AD	Significant effects for 150 mg/day as compared with placebo:BPD severity self-rated (BEST), SMD − 0.67, *p* = 0.01; BPD severity interviewer-rated (Zan-BPD): SMD − 0.55, *p* = 0.03), psychosocial functioning (SDS), SMD − 1.36, *p* < 0.0001; aggression (OAS-M) SMD − 0.65, *p* = .01; non-serious adverse events, RR 1.34, *p* = .05)	Significant effects for 300 mg/day as compared with placebo, BPD severity self-rated (BEST), SMD − 0.57, *p* = .02; psychosocial functioning (SDS), SMD, *p* < .00001; aggression (OAS-M), SMD − 0.60, *p* = .02; mania (YMS), SMD − 1.21, *p* < .0001; non-serious AE, RR 1.39, *p* = .02
					Lee et al. 2016 [14]: SCL-90-R subscales	Significant effects for 150 mg/day as compared with placebo,interpersonal sensitivity (*d* = − 0.58, *p* = 0.038, depression *d* = − 0.87, *p =* 0.007), hostility (*d* = − 0.71, *p* = 0.017)	Significant effects for 150 mg/day as compared with placebo,interpersonal sensitivity (*d* = − 0.80*, p* = 0.05), depression (*d* = − 0.94, *p* = 0.04), hostility (*d* = − 0.68, *p* = 0.023), phobic anxiety (*d* = −0.66, *p* = 0.023), SCL-90-R total (*d* = − 0.62, *p* = 0.033)
Mood stabiliser							
Crawford et al. 2018 [12, 15]	*n* = 195 participants with BPD (24.6% men)	Lamotrigine (up to 200 mg/day)	Placebo	52 weeks	BPD symptoms, self-harm, social functioning, drug and alcohol use, health-related quality of life, AE, and costs	No significant difference was observed for any outcome	
Other substance classes							
Kulkarni 2018 [13]	*n* = 18 participants with BPD (13.8% men)	Memantine (anti-dementia drug; up to 20 mg/day) as adjunct to ongoing psychotherapy and/or medication	Placebo	8 weeks	BPD symptoms, AEs	Significantly higher rate of change in BPD severity (latent growth curve analysis; *b* = 7.30, *p* = 0.02) reported by study authors; between-group effects do not indicate a significant difference SMD 0.37 (− 0.32, 1.06)	
NCT01212588 [20]	*n* = 22 participants with BPD (13.5% men)	Mifepristone (3 × 200 mg/day)	Placebo	7 days	BPD pathology, general psychopathology, psychotic symptoms, and AE	No significant effects for mifepristone, instead consistent trend of better outcomes in placebo group, including one sig. effect indicating less identity disturbance in control group (SMD 0.97, 95% CI 0.08 to 1.87)	
NCT00539188 [21]	*n* = 6 participants with BPD (33% men)	N-Acetylcysteine3000 mg PO (1200 mg a.m., 1800 mg p.m.),	Placebo	6 weeks	Self-harm	Study was withdrawn due to poor subject compliance.	

*AE* adverse events; *BEST* Borderline Evaluation of Severity over Time; *d* mean change compared with placebo mean change from baseline, divided by pooled baseline standard deviation; *DBT* Dialectical Behaviour Therapy, *i.v.* intravenous, *n.s.* not significant, *OAS-M* Overt Aggression Scale-Modified, *RR* Risk Ratio, *SCL-90-R* Symptom Checklist-90-Revised, *SDS* Sheehan Disability Scale, *SMD* standardised mean difference, *SP* supportive psychotherapy, *YMS* Young Mania Scale, *Zan-BPD* Zanarini rating scale for borderline personality disorder

### Antidepressants

#### Recently Published RCT Data

We did not identify any new full publication of a controlled study or RCTs testing antidepressants in people affected by BPD. Following up the studies which we had listed as ongoing in our previous review [10], we found data posted on ClinicalTrials.gov for two antidepressant RCTs [18•, 19•]. Both these trials investigated fluoxetine. From our calculation of available data, we did not find significantly different rates of serious adverse events (AE) if fluoxetine was compared with placebo [19•]. In contrast, from the RCT, which compared fluoxetine to Dialectical Behavioural Therapy [18•], we observed a significantly higher rate of suicide attempts in the medication-only group (Table 1).

#### Unpublished RCTs

When following up the references that we had identified as ongoing in our 2015 review, we found that three were still unpublished: One RCT of escitalopram had never been started [22], whereas another RCT of escitalopram was terminated because of difficulties in recruiting participants [23]. A third RCT testing the MAO-I selegiline against placebo was completed, but though our best efforts, we were not able to find any corresponding publication [24]. The latter was the only industry-sponsored trial among the listed unpublished studies.

#### Ongoing RCTs

We did not identify any ongoing RCT evaluating the effects of continued antidepressant treatment.

#### Critical Judgement of Current Evidence

Given the high rates of antidepressant use in BPD, the lack of relating evidence is startling. The most recent placebo-controlled RCT testing an antidepressant is still the one of Simpson and colleagues, published in 2004 [25]. Large cohort studies concurrently show that antidepressants are the medication class which is most often prescribed, with rates ranging between 70 and 80% [8, 26–28]. Of course, depressive disorders are highly prevalent in BPD, and if a manifest depressive disorder is present, it may require medication. Yet, another study which compared the medication rates of those inpatients with BPD who had a comorbid depressive disorder to the medication rates of those who had not did not find a difference in the type and number of medications [29]. Paton and colleagues report antidepressant use in 94% of patients with an emotionally unstable personality disorder (EuPD) who also had comorbid depression, but also in 75% of people who had EuPD as their sole psychiatric diagnosis [27]. Obviously, comorbidity does not account for the high use of medication in BPD. Despite the complete lack of reliable, supporting evidence, be it from meta-analytically accumulated evidence or at least single RCTs, antidepressants are still prescribed “by default”, it seems [30].

### Antipsychotics

#### Recently Published RCT Data

We were able to identify one new RCT which compared the effects of the two second-generation antipsychotics (SGAs) olanzapine and asenapine [11•], and two records [14•, 17•] adding new data to a yet published placebo-controlled RCT of quetiapine [16•] (s. Table 1). Bozzatello and colleagues compared olanzapine and asenapine head to head and observed very limited differences: olanzapine was superior regarding dissociation, asenapine regarding affective instability. Unfortunately, the trial did not include a placebo control group, so this trial compared two substances of uncertain efficacy. To date, we cannot conclude from the evidence that olanzapine was a helpful and safe treatment option [10, 31], and asenapine has never been subject to a placebo-controlled RCT in BPD so far. Even if asenapine had been found to be convincingly superior to olanzapine, it would still be uncertain if patients would profit at all. Given the limited therapeutic effects on the one hand and the well-documented adverse effects of both substances that were also observed within this trial (asenapine: oral hypoesthesia, anxiety/restlessness, akathisia; olanzapine: weight gain, somnolence, fatigue) on the other, neither of the two options seems to be a treatment option.

New data have become available for the only one existing placebo-controlled RCT of quetiapine [16•]. The authors posted final raw data at the ClinicalTrails.gov website [17•], which allow for calculating between-group effect sizes at the time of post-treatment. Using these data, we observed moderate to large, statistically significant effects for both doses of quetiapine (150 mg/day and 300 mg/day) regarding BPD severity, psychosocial impairment and aggression, and an additional effect for the higher dose regarding manic symptoms (s Table 1). Comparing the two active groups, we found no superiority of any group. Notably, there was no clear dose-effect relationship for clinical outcomes, i.e. beneficial effects of treatment, but a higher proportion of participants experiencing adverse events in the group with the higher dose. Both doses were associated with significantly heightened rates of adverse events, the most prevalent ones being sedation, mouth dryness, change in appetite and headache. These newer data support the previously reported findings published in the 2014 paper [16•].

#### Ongoing Studies

Two placebo-controlled RCTs are currently underway to evaluate the effects of brexpiprazole [32, 33], an SGA which has been described as a serotonin–dopamine activity modulator (SDAM). It has been approved for the treatment of schizophrenia by the Food and Drug Administration (FDA) and by the European Medicines Agency (EMA), but it is additionally approved as an adjunctive to antidepressants in major depressive disorder treatment in the USA. Another placebo-controlled RCT is underway in the UK where inpatients with BPD who did not experience an adequate clinical response to antipsychotic medications will receive clozapine [34]. Last, we are aware of another placebo-controlled RCT of aripiprazole for people with a diagnosis of BPD and auditory verbal hallucinations, but without schizophrenia or any psychotic disorder [35]. Notably, this will be one of the first RCTs ever investigating the beneficial and harmful effects of pharmaceutical treatments in adolescents with BPD (minimum age, 15 years; maximum age, 25 years). For an overview of ongoing studies, see Table 2.

**Table 2:** Currently ongoing randomised-controlled trials identified in ClinicalTrials.gov, EudraCT, ICTRP and ISRCTN (April 2020)

Trial registration number	Participants	Sponsor	Registration date (month/year)	Interventional drug	Comparison treatment	Observation period	Clinical outcomes
Antipsychotics							
ACTRN12616001192471 [35]	BPD + auditory verbal hallucinations, aged 15 to 25 years	N/a	08/2018	Aripiprazole (up to 30 mg/day)	Placebo	12 weeks	Severity of auditory verbal hallucinations, BPD severity, depression, anxiety, psychotic symptoms, and psychosocial functioning
ISRCTN18352058 [34]	Inpatients with BPD without adequate clinical response to antipsychotic medication other than clozapine	University	03/2019	Clozapine (up to 400 mg/day)	Placebo	6 months	BPD severity, psychotic symptoms, suicidal behaviour, aggression, health-related quality of life, side effects, medication adherence, and service use
NCT03418675 [32]	BPD	University, industry	02/2018	Brexpiprazole (up to 2 mg/day)	Placebo	12 weeks	BPD severity, aggression, impulsiveness, suicidality, anxiety, depression, impairment, quality of life, and mania
NCT04100096 [33]	BPD	Industry	09/2019	Brexpiprazole (up to 3 mg/day	Placebo	12 weeks	BPD severity, global clinical severity
Explanatory trials miscellaneous							
NCT03395314 [45]	BPD	University	01/2018	Ketamine i.v. 0.5 mg/kg over 40 min	Placebo (midazolam, 0.04 mg/kg over 40 min)	Single application, observation period 4 weeks	BPD severity, suicidality, depression, anxiety, psychosocial functioning, and psychotic symptoms
NCT02728778 [46]	BPD	University	04/2016	Botulinum toxin A Single administration of incobotulinumtoxin A into the forehead (glabellar region); 34 U in five injection sites	Acupuncture into the forehead	Single application, observation period 16 weeks	BPD pathology, depression

*ACTRN* Australian Clinical Trials Registration Number, *EudraCT* European Union Drug Regulating Authorities Clinical Trials Database, *ICTRP* International Clinical Trials Registration Platform, *ISCTN* International Standard Randomised Controlled Trial Number

#### Critical Judgement of Current Evidence

International studies concordantly report antipsychotics being prevalently prescribed to people with a diagnosis of BPD, with rates ranging between 70 and 79% of inpatients [8, 29, 36] and 35 and 60% of outpatients [26–28]. Given the current evidence, it takes wonder why quetiapine plays such an outstanding role within BPD treatment. Among antipsychotic agents, quetiapine is the one which is most often described to people with a diagnosis of BPD, and, above all, it is also the one single substance most often described among all substance classes: actually, every fifth to almost third inpatient would receive quetiapine [8, 29, 36]. To date, only one single RCT [14•, 16•, 17•] has ever been published that evaluated the effects of this substance in people with BPD. At the same time, we are aware of a number of RCTs involving quetiapine that have been completed, but results have not been published [37, 38]. Another RCT involving the antipsychotic risperidone has been registered and was recruiting, but we do not know if this trial has ever been completed, and if so, what the results were [39]. In our previous Cochrane review [31], we had included eight placebo-controlled RCTs of second-generation antipsychotics (SGAs; aripiprazole, olanzapine, ziprasidone), but did not observe robust evidence of clinically relevant effects for any of the substances. Instead, we found significant adverse effects for olanzapine, including metabolic changes and weight gain, and indications of more self-harming behaviour under olanzapine. Since our Cochrane review in 2010 [31, 40], only one placebo-controlled RCT of any SGA has been published [16•], indicating some effects of quetiapine regarding psychosocial functioning, BPD severity, and aggression, but also significant adverse events.

### Mood Stabilisers

#### Recently Published RCT Data

A large RCT study has been conducted by Crawford and colleagues [12••, 15••]. They compared the effects of 12 months of lamotrigine treatment to placebo and observed not one significant difference between the groups. Including a large sample of 276 participants, the study had enough power to detect clinically relevant differences, had there been any. Other strengths of this study are the long observation period (whereas most pharmacotherapy trials last for 12 weeks only on average [31]), and the broad inclusion criteria (only comorbid bipolar and psychotic disorders were excluded). The authors conclude that lamotrigine is neither a clinically nor a cost-effective treatment option for BPD.

#### Ongoing Studies

Notably, we were not able to identify any ongoing study of mood stabilisers for the treatment of BPD.

#### Critical Judgement of Current Evidence

Previously, mood stabilisers had been reputed a possible alternative role to antipsychotic agents [41, 42]. In our previous Cochrane review from 2010, we also observed some effects for the mood stabilisers lamotrigine, topiramate, and valproic acid [31, 40], and the meantime publication of another, small RCT [43] did not change the results substantially [10]. However, the underlying studies were small, with samples ranging between 15 and 56 participants, included short observation periods and partly applied strict exclusion criteria. Therefore, the new findings of lamotrigine treatment in routine care shed a completely new, more critical light on the use of mood stabilisers. Weighing uncertain beneficial against possible adverse (i.a. teratogenic) effects, mood stabilisers are not an expedient treatment option in a population of patients, most of which are women in childbearing age.

### Explanatory Trials

#### Recently Published RCT Data

Memantine, a substance anti-dementia drug that targets the glutamatergic system, has been tested against placebo [13•]. Many people with BPD have chronically been exposed to stress due to maltreatment during childhood. Chronic stress can induce changes in glutamate release and glutamate receptor functioning, resulting in overactivity and excitotoxicity. By moderating glutamatergic neurotransmission, the NMDA antagonist memantine may reduce BPD severity, and self-harming and impulsive behaviour specifically, which all have been found to be correlated to glutamate concentrations [44]. The study authors report a significant higher rate of change of BPD severity by memantine in a latent growth curve analysis. Calculating the SMD from post-treatment group data, we could not replicate a significant effect (SMD 0.37 (− 0.32, 1.06), *p* = .30). Instead, the final BPD severity score was higher in the memantine group.

We identified another placebo-controlled RCT without full-text publication, but with outcome data posted at the ClinicalTrials.gov registry [20•]. In this study, the effects of mifepristone, a glucocorticoid receptor antagonist that is also used as abortifacient, are tested. Mifepristone is supposed to alter a putative hyper-responsiveness of the hypothalamic-pituitary-adrenal (HPA) axis. Calculating between-group effects at post-treatment from the posted raw data, we did not find any statistically significant effect favouring mifepristone, but a consistent trend throughout all reported measures of better results in the placebo group. These findings speak against a possible role for mifepristone in BPD treatment.

Last, we found a reference relating to a small placebo-controlled study of *N*-Acetylcysteine (ACC) as an adjunct to an ongoing Dialectical Behavior Therapy (DBT) [21•]. However, the posted study results refer to six participants, of which two completed the 6-week study (one in each group). The authors state that the study was terminated due to poor subject compliance.

#### Ongoing Studies

We are aware of an ongoing RCT testing the effects of ketamine against a control treatment with the relaxant midazolam [45]. Ketamine has been observed to rapidly reduce suicidality and improve mood in patients with MDD. This study aims to assess if these effects can also be found in people with a diagnosis of BPD.

Last, the effects of applicating botulinum toxin A, a relaxant that is injected into the forehead, are studied in an ongoing RCT [46]. Application of botulinum toxin A results in a paralysis of facial muscles that are involved in the expression of negative emotions. Thus, the afferent feedback is interrupted, which putatively alleviates depressive symptoms. Participants allocated to the control condition will receive acupuncture sessions. Outcome assessment includes depression as well as BPD pathology.

## Conclusions

As in our 2015 review, we must conclude that the evidence of drug treatment effects has not accumulated substantially, a finding which is almost identical to our conclusion 5 years ago. However, the new findings of no beneficial effects by fluoxetine in the prevention of suicidal behaviour in people with BPD (but inferiority to psychotherapy treatment instead) merit closer attention. SSRIs being among the most often used substances in BPD, regardless of co-existing comorbidities, it is remarkable that only three placebo-controlled RCTs of SSRIs in BPD exist, involving, altogether, data of 85 participants [25, 47, 48].

Another remarkable new finding is the failure to detect beneficial effects by lamotrigine treatment in a real-world effectiveness study [12••, 15••]. This large-scale pragmatic RCTs yield conclusive results that are applicable routine care, suggesting that lamotrigine is not a clinically effective treatment for people with BPD.

The drug industry has retracted from initiating large-scale trials in recent years. Eli Lilly had sponsored two large-scale RCTs of olanzapine [49, 50], which included more than 300 participants each, but did not result in drug approval for use in BPD. After that, there were no major industry-funded research efforts. Medication use being still off-label in BPD, drugs are nevertheless extensively prescribed to people with BPD. The lack of conclusive supporting evidence seems not to hinder the use of medications in routine care. For example, quetiapine has been found to be the single substance most often described to people with BPD at a time when not a single placebo-controlled RCT had been available that would have evaluated the effects of quetiapine in BPD at all [8]. Furthermore, comorbidities like affective, anxiety or traumatic disorders are common in BPD [51], and these conditions may warrant medication use, which is in line with recommendations of the major BPD guidelines [1, 2]. However, data from clinical practice indicate that drug use is not related to comorbidity [27, 29].

We are facing a manifest gap between evidence and practice. In order to enable people affected by BPD as well as clinicians to make informed decisions, more RCTs are needed. On the one hand, these should replicate previous research where positive findings were observed in single, small studies [31, 40]. On the other hand, under-researched areas need to be approached: Medication use (especially SSRI) is oftentimes justified by the prevalence of comorbid disorders, especially depression, but there is a clear lack of studies that focused on participants with distinct comorbidities. In terms of interventions, we need more RCTs evaluating the highly used drugs like SSRIs, and quetiapine. Fortunately, there are first new efforts from the industry to evaluate new substances in explanatory trials [32, 33]. As long as there is no definite conclusion about any substance being effective, trials should include a placebo comparison group. Outcome assessment should include BPD-specific pathology, but also pathology related to comorbid conditions if comorbid samples are in focus. Psychosocial functioning has been found to be crucial for judging mid- and long-term effects [52] and should also be assessed. Adverse events should regularly be monitored in a standardised way. Last, observation periods should last sufficiently long to reflect clinical practice and to allow for drawing conclusions about beneficial effects. Our working group is currently working on an update of the 2010 Cochrane review [53]. Based on comprehensive searches, we intend to review and statistically integrate the available evidence. In this Cochrane review, we will also include a quality assessment of the included trials which we have not done in this review.
